# On the understanding of current-induced spin polarization of three-dimensional topological insulators

**DOI:** 10.1038/s41467-019-09271-1

**Published:** 2019-04-01

**Authors:** Jifa Tian, Seokmin Hong, Shehrin Sayed, Joon Sue Lee, Supriyo Datta, Nitin Samarth, Yong P. Chen

**Affiliations:** 1 Department of Physics and Astronomy, Purdue University, West Lafayette, IN 47907 USA; 2 Birck Nanotechnology Center, Purdue University, West Lafayette, IN 47907 USA; 3 Department of Physics and Astronomy, University of Wyoming, Laramie, WY 82071 USA; 4 School of Electrical and Computer Engineering, Purdue University, West Lafayette, IN 47907 USA; 5 Center for spintronics, Post-silicon Semiconductor Institute, Korea Institute of Science and Technology (KIST), Seoul, 02792 South Korea; 6 Electrical Engineering and Computer Science, University of California Berkeley, CA, 94720 USA; 7 Department of Physics, The Pennsylvania State University, University Park, Pennsylvania, 16802 USA; 8 Purdue Quantum Science and Engineering Institute, Purdue University, West Lafayette, IN 47907 USA; 9 WPI-AIMR International Research Center for Materials Sciences, Tohoku University, Sendai, 980-8577 Japan

**Keywords:** Electronics, photonics and device physics, Electronic and spintronic devices, Quantum physics

**Arising from** C. H. Li et al. *Nature Communications* 10.1038/ncomms13518 (2016).

In a recent article^1^, Li et al. reported the current-induced spin polarization (CISP) on topological insulator (TI) Bi_2_Se_3_ and on InAs (100) samples by spin potentiometry and compared the sign of the measured signals using a theoretical model to conclude the origin of the observed CISP in their TIs. Spin potentiometry has been used to electrically measure CISP in spin-orbital coupled (SOC) materials, such as TIs^2–8^, and semiconductor-based two-dimensional electron gases (2DEG)^9–11,12^, where a ferromagnetic (FM) voltage probe with magnetization (**M**) collinear to the induced non-equilibrium spins is used to determine the CISP. The theoretical model by Li et al. consists of two key components: (a) a spin-dependent electrochemical potential diagram and (b) an argument that FM probe with magnetization along up (down) spin direction will measure the down (up) spin electrochemical potential. However, we bring attention to the inconsistencies in their paper. First, their (spatially varying) spin-dependent electrochemical potential diagram is incorrect and inconsistent with their topological surface states (TSS) band diagram to reflect the same experimental condition. The corrected potential diagram in conjunction with their model argument (b) is inconsistent with their assigned origin of CISP based on the sign of the measured signal. Second, they incorrectly stated that the experiment on (Bi,Sb)_2_Te_3_ reported by Lee et al.^7^ gave the same sign as their measurements, whereas it is opposite. Finally, we point out that the comparison of the sign of the measured signal from TI with that of InAs may not be sufficient to draw a conclusion on the origin of CISP in their measurements.

Li et al.^1^ adopted a model similar to our prior model^5,13,14^ to explain their observed CISP in terms of the spin-momentum locking (SML) of TSS (see Fig. 1). However, there is a qualitative difference regarding the electrochemical potentials between the model described by Li et al. in ref. ^1^ (Fig. 1b, c taken from Fig. 5 of ref. ^1^) and our model (Fig. 1d, e). First, their electrochemical potential diagram (see Fig. 1c) is inconsistent with their band diagram (see Fig. 1b) which is supposed to reflect the same experimental situation (see Fig. 1a). In Fig. 1c, the chemical potential of up-spin (*μ*_↑_) is lower than that of down-spin (*μ*_↓_), whereas *μ*_↑_ is higher than *μ*_↓_ in Fig. 1b. The relative order and the magnitude of the two opposite spin-dependent electrochemical potentials should be self-consistent between the band diagram (energy versus momentum) and their spatial distribution plot (energy versus distance), namely *μ*_↑_ should be higher than *μ*_↓_ in both Fig. 1b, c. We have drawn the corrected versions of Fig. 1b, c in Fig. 1d (band diagram) and Fig. 1e (spatial variation), respectively, under the same bias condition as Fig. 1b, c. Furthermore, Li et al.^1^ used the absolute values of the electrochemical potentials (|*μ*_↑_| and |*μ*_↓_|) in the band diagram (see Fig. 1b), which is also incorrect since it changes the actual physics depending on the choice of the ground or zero energy level. Generally speaking, which spin state of electrons is more occupied should be governed by the difference (*μ*_↑_ − *μ*_↓_) between *μ*_↑_ and *μ*_↓_, not by their absolute values. Moreover, unlike what is depicted in Fig. 1c, the choice of the reference position for zero potential (*µ* = 0) is not important for determining which spin state is more occupied (or which spin chemical potential is higher). Since the electrons are injected by the right contact and flow from right to left in Fig. 1c, the chemical potential (*μ*_R_) of the right contact is higher than that (*μ*_L_) of the left contact (see Fig. 1c). Meanwhile, this also means that in the channel there should be more occupation of the left-going electron states (corresponding to up-spin states in a TI channel with the SML of TSS) than the right-going states (corresponding to the down-spin states), thus *μ*_↑_ (equivalently the chemical potential of left-going electrons) should be higher than *μ*_↓_ (the chemical potential of right-going electrons), whereas *μ*_↑_ is incorrectly drawn to be lower than *μ*_↓_ in Fig. 1b. Based on the above arguments, the sign of the spin voltage expected from the corrected potential diagram (seen Fig. 1d, e) in conjunction with their model argument b is opposite to the signal sign on Bi_2_Se_3_ reported by Li et al. Thus, the origin of the CISP observed in their Bi_2_Se_3_ samples is inconsistent with the expected CISP from the TSS.

**Fig. 1:**
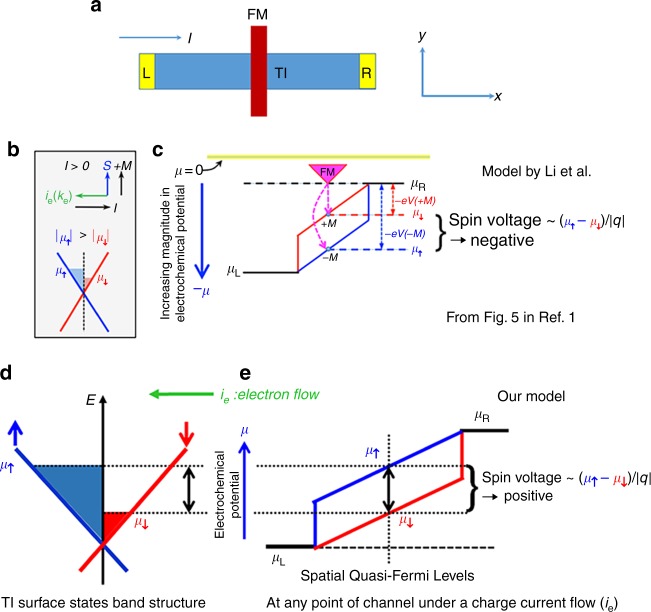
Comparison of the models depicting the sign of the spin signals expected from TSS. **a** The schematic structure of a device with a charge current (I) flowing from left (L) to the right (R); **b** The band structure diagram of TSS, taken from the left panel shared by Fig. 5b, c in ref. ^1^; **c** Diagram showing spatially varying spin-dependent electrochemical potential, taken from the top panel of Fig. 5c in ref. ^1^. **d**, **e** Our understanding of the spin-dependent electrochemical potentials of TSS in 3D TIs under the same bias current as that shown in **b**, **c**. The chemical potential (*μ*_↑_) of up spins is higher than that (*μ*_↓_) of down spins in both **d** and **e**

We further note that Li et al.^1^ incorrectly stated that another experiment on (Bi,Sb)_2_Te_3_ by Lee et al.^7^ (which is ref. 29 in ref. ^1^) gave a consistent sign as theirs, whereas it is opposite. We point out that the sign reported by Lee et al.^7^ is consistent with several other reports (see, refs. ^5,6,8^) while opposite to Li et al.^1,2^, as summarized in Table 1.

**Table 1:** Comparison of the reported results on CISP in 3D TIs measured by spin potentiometry

	Li et al. (refs.^1, 2^)	Tian et al. (ref. ^5^)	Dankert et al. (ref. ^6^)	Lee et al. (ref. ^7^)	Yang et al. (ref. ^8^)
Charge current direction (*I*_c_)	+x	+x	+x	+x	+x
Electron current direction (*I*_e_)	−x	−x	−x	−x	−x
Magnetization direction of FM (M)	+y	+y	+y	+y	+y
Sign of spin signal (*V*_S_)	−	+	+	+	+
Channel spin polarization direction (*s*_c_)	−y	+y	+y	+y	+y
Spin polarization from TSS (*s*)	+y	+y	+y	+y	+y

Li et al.^1^ also attempted to infer the origin of CISP in their Bi_2_Se_3_ by making a comparison with SOC semiconductor InAs, where a Rashba-type 2DEG normally exists on the surface. We caution that such a comparison may not be sufficient to draw a conclusion on the origin of CISP. For example, it is known that, depending on the direction of the effective electric field (potential gradient) perpendicular to the 2DEG^9–11,12^, the spin helicity of the outer Rashba band (which dominates the signals measured in transport) can be either opposite to or the same as that of TSS. Without a careful consideration of various parameters (e.g., an independent determination of the spin texture, for example by ARPES, and consideration of capping surfaces, interfaces, etc.) of their samples, spin potentiometric measurements in their InAs sample cannot provide an unambiguous “calibration” to determine the direction of the CISP measured by spin potentiometry in their Bi_2_Se_3_.

Further complications can arise from the fact that in addition to the nontrivial spin-momentum-locked TSS, Bi_2_Se_3_ often contains multiple bands and conducting channels with spin-orbit coupling that can affect CISP. For example, the trivial surface 2DEG derived from bulk states and often observed by ARPES^15^ in Bi_2_Se_3_ possesses strong Rashba-type spin-orbit coupling and typically has two fermi surfaces with opposite spin helicities^12^. We note that the model (Fig. 1d, e) we developed is only for TSS in the ideal case of bulk-insulating TI materials where the Fermi level is inside the bulk band gap. If the TI samples have metallic bulk with their Fermi levels located in the conduction band where the multiple bands coexist, our model may not be sufficient to determine the sources of the measured CISP.

In conclusion, the model used by Li et al.^1^ is erroneous and inconsistent with their TSS band diagram, and also inconsistent with the expected CISP due to TSS. The sign of the CISP signal experimentally observed in their Bi_2_Se_3_ is opposite to that predicted by a corrected model based on TSS and that observed in other experiments on various TI materials^5–8^. Owing to the existence of multiple bands in the bulk-metallic TI samples, the source of the measured CISP can be complicated, possibly involving competition between different bands (e.g., TSS and Rashba bands). Identifying the origin of CISP in bulk-metallic TI samples such as the Bi_2_Se_3_ used by Li et al.^1^ may require analysis beyond the simple model (Fig. 1c, d) we developed.

## Data Availability

All relevant data are available from the authors upon request.
